# Unfavorable management outcome and its predictors among appendicitis patients in ethiopia: a systematic review and meta- analysis

**DOI:** 10.1186/s12893-025-03108-z

**Published:** 2025-08-09

**Authors:** Mulualem Gete Feleke, Tadele Lankrew Ayalew, Kidist Ashager, Hailu Asmare Beyene, Moges Wubneh Abate, Nigusie Selomon Tibebu

**Affiliations:** 1 School of Nursing, College of Medicine and Health Sciences, Wolaita Sodo University, Wolaita Sodo, Ethiopia; 2 Departments of Adult Health Nursing, College of Health Sciences, Debre Tabor University, Debre Tabor, Ethiopia; 3 Departments of Pediatrics and Child Health Nursing, College of Health Sciences, Debre Tabor University, Debre Tabor, Ethiopia

**Keywords:** Appendicitis, Unfavorable, Management outcome, Factors, Ethiopia

## Abstract

**Background:**

Appendicitis is life-threatening abdominal surgical emergency worldwide, requires timely medical intervention to prevent adverse outcomes such as wound infection, pneumonia, intra-peritoneal fluid collection, and death. These complications remain a significant challenge in Ethiopia. This study aimed to assess the pooled magnitude of unfavorable management outcome and identify associated factors among appendicitis patients in Ethiopia.

**Methods:**

We conducted a systematic search of databases for studies reporting appendicitis outcomes in Ethiopia. Eligible studies were screened based on predefined criteria. Statistical analyses were performed using STATA version 14. Heterogeneity was assessed with I² and Cochran’s Q tests; due to substantial heterogeneity, a random-effects model was applied. Publication bias was evaluated using funnel plots, Egger’s test, and the trim-and-fill method. Subgroup analyses explored sources of heterogeneity based on study region and hospital type.

**Results:**

Eighteen studies articles were included. The pooled prevalence of unfavorable management outcome among appendicitis patient was 12.71% (95%CI: 9.32–16.09). Sub-group analysis showed that Oromia region had the highest prevalence of poor management outcome. Duration of illness [AOR = 4.41 (95%CI: 1.42–13.70)], right lower quadrant abdominal mass [AOR = 4.1 (95%CI: 2.29–7.34)], presence of intraoperative abscess [AOR = 6.9 (95% CI: 3.61–13.22)], lengths of postoperative hospital stays [OR = 5.28 (95%CI: 2.31–12.04)], elevated white blood cell count [AOR = 4.09 (95%CI: 2.22–7.54)] were a significant association with unfavorable management outcome of appendicitis.

**Conclusion:**

Unfavorable management outcomes in appendicitis patients in Ethiopia are significantly associated with several clinical factors. Enhancing early diagnosis, prompt surgical intervention, and standardized postoperative care are essential to reduce these adverse outcomes.

## Introduction

Appendicitis, characterized by the inflammation of the vermiform appendix, is a common and potentially life-threatening abdominal surgical emergency, If not promptly treated; it can lead to serious complications such as ileus, peritonitis, abscess formation, and death, resulting significant morbidity and mortality ae well as a substantial financial burden on healthcare systems globally [1–3]. Despite advancements in medical and surgical care, it remains a prominent global health issue, contributing to a high volume of emergency surgical admissions and substantial healthcare costs [4–7].

Early diagnosis and standardized treatment protocols are critical to improving outcomes. International evidence-based guidelines such as those from the World Society of Emergency Surgery (WSES) and the Society of American Gastrointestinal and Endoscopic Surgeons (SAGES) emphasize the importance of early clinical evaluation, timely imaging, and prompt surgical or non-operative management to minimize complications and mortality [8, 9].

The global incidence of appendicitis is approximately 233 per 100,000 populations per year, with a lifetime incidence risk ranging from 6.7 to 8.6% [10]. Acute appendicitis is the most prevalent in low-income countries [11]. The prevalence of acute appendicitis was different across the country: a study conducted in western Sudan showed that the prevalence rate was 63% [12], 22.1–49.8 in Nigeria [13] and 46.95% in Ethiopia [14]. In Ethiopia, the magnitude of unfavorable outcome among appendicitis patients varies widely, ranging from 1.4%-31.9%% in different studies [15–26],

Unfavorable management outcomes in appendicitis patients are influenced by various clinical and demographic factors. The most common unfavorable outcomes of appendicitis were wound infection [27, 28], pneumonia [29], intra-peritoneal fluid collection [30] and death [27, 30] serve as indicators of the quality of surgical services within a healthcare system. Evidence showed that the overall prevalence of unfavorable management outcome following appendectomy ranged from 8.2 to 31.4%, with wound infection rates between 3.3 and 10.3% and pelvic abscess incidence around 9.4% [31, 32]. Several predictors has been associated with unfavorable management outcome, including fever [19], gender [21], delayed presentation to the hospital [15, 16, 19, 21, 33], prolonged hospital stay [18, 19, 21, 25, 33, 34], presence of mass in the right lower quadrant abdomen [17, 18, 20, 21, 25], elevated white blood cell count [22, 23], and intraoperative findings of appendicular abscess or perforation [15, 17–19, 23, 25, 33, 35].

Evidence from other low- and middle-income countries (LMICs) suggests that resource limitations such as inadequate diagnostic facilities, limited surgical infrastructure, and inconsistent perioperative care contribute to higher complication rates compared to high-income countries [36–38]. In Ethiopia, findings across regions remain inconsistent, and no comprehensive synthesis has been undertaken to evaluate the pooled prevalence and predictors of unfavorable outcomes. This systematic review and meta-analysis aims to determine the pooled prevalence of unfavorable treatment outcomes and its associated factors among appendicitis patients in Ethiopia. The findings will provide valuable insights into the epidemiological and clinical contributors to these outcomes, which could inform policy development and improve healthcare delivery in the region. The existing literature highlights the importance of early diagnosis and intervention in improving treatment outcomes to reduce complications and improve patient’s prognosis.

## Methods and materials

This systematic review and meta-analysis was conducted in accordance with the PRISMA (Preferred Reporting Items for Systematic Reviews and Meta-Analyses) 2020 guidelines guideline [39]. Although the protocol was not prospectively registered in PROSPERO, it was developed prior to data extraction and adhered to rigorous methodological standards throughout the review process.”

### Search strategy and review process

A comprehensive search was conducted in major biomedical databases including PubMed, Google Scholar, EMBASE, HINARI, and the Cochrane Library. Grey literature such as theses and dissertations was also reviewed to minimize publication bias. Search terms were combined using Boolean operators (“AND”, “OR”) and included: (magnitude OR prevalence OR proportion) AND (favorable OR unfavorable) AND (treatment outcome OR management outcome) AND (associated factor OR predictors OR determinants) AND (appendicitis) AND (Ethiopia). Studies published up to March 2025 were considered. Additionally, reference lists of relevant studies were screened to identify additional eligible articles. The inclusion of gray literature helped enhance the comprehensiveness of the review by incorporating regional and unpublished data, although all sources were carefully assessed for relevance and quality. Data extraction and critical appraisal procedures were applied uniformly across both published and gray literature sources to ensure consistency.

## Eligibility criteria

### Inclusion criteria

#### Inclusion and exclusion criteria

Studies were included if they met the following criteria:

- Conducted in Ethiopia.
- Reported on management outcomes and/or associated factors in appendicitis patients.
- Articles written in English language.
- Employed an observational design (cross-sectional or cohort).
- Available as full-text articles, theses, or dissertations Studies from the same institution conducted in the same year but with non-overlapping data collection periods were both included, assuming distinct participant groups without duplication.

Studies lacking critical data, presenting methodological flaws, or failing to provide missing information despite a four-week follow-up with corresponding authors were excluded.

### Study selection and data extraction

All identified articles were managed using EndNote version 21. Following duplicate removal, three reviewers independently screened titles and abstracts for relevance. Full-text screening was subsequently performed by two reviewers, with disagreements resolved through discussion. data extraction was performed using standardized form, which capturing key variables such as author, publication year, study region, sample size, hospital type, prevalence of unfavorable outcomes, types of unfavorable management outcome, and associated predictors including age, sex, duration of illness, length of hospital stay, elevated white blood cell (WBC) count, and intraoperative findings (perforation and abscess). For this review, “perforation” was defined as a transmural rupture of the appendix identified intraoperative, while “intraoperative abscess” referred to a localized collection of pus observed during surgery. These factors were evaluated as predictors or determinants of unfavorable outcomes. The extraction process was conducted independently by two reviewers, with discrepancies resolved through discussion or consultation with a third reviewer to ensure accuracy.

### Outcome measurements

The primary outcome was the pooled prevalence of unfavorable management outcomes among appendicitis patients in Ethiopia. Unfavorable management outcomes, as reported in the primary studies, were defined as the occurrence of one or more postoperative complications, including but not limited to fecal fistula, pneumonia, wound dehiscence, mortality, ileus, wound infection, sepsis, hernia, and intra-abdominal fluid collection. The pooled prevalence reflects the proportion of patients experiencing at least one of these unfavorable outcomes. The magnitude was determined by dividing the number of participants with unfavorable management outcome by the total number of appendicitis patients in the study (sample size) and multiplying the result by 100. The secondary outcome was the identification of predictors significantly associated with these outcomes. Only predictors reported in two or more studies were considered.

### Quality assessment

The methodological quality of the included studies was assessed using the Newcastle–Ottawa Scale (NOS) adapted for cross-sectional studies [40]. This tool assesses studies across three key domains:

1. Methodological Quality (up to 5 stars), including sample representativeness, sample size, and outcome ascertainment;
2. Comparability (up to 2 stars), based on control for confounding factors; and.
3. Outcome (up to 3 stars), Based on outcome assessment, statistical test quality, and response rate.

The NOS assesses selection, comparability, and outcome domains, with a total possible score of 10 stars. Studies scoring ≥ 6 out of 10 were considered of high quality. Two reviewers independently assessed study quality. Any disagreements were resolved through discussion until consensus was reached. All included studies met the minimum quality threshold and were retained for analysis.

The methodological quality of the included studies was moderate to high, with NOS scores ranging from 6 to 9. A total of 17 studies (94.4%) were rated as high quality (scores ≥ 7), while one study (5.6%) was rated as moderate quality (score = 6). The selection domain was consistently strong, with 88.9% of studies scoring 4 out of 5, indicating appropriate sampling methods and outcome ascertainment. The comparability domain was also well addressed, with 83.3% of studies achieving the maximum score, reflecting adequate control for confounding variables. In the outcome domain, all studies scored at least 2 out of 3, and 38.9% attained full scores, demonstrating the use of appropriate statistical analyses and outcome measurements. No studies were excluded based on quality, as all met the minimum inclusion threshold. The full NOS quality assessment is summarized in (Table 1).

**Table 1:** NOS quality assessment of included studies on unfavorable management outcome and associated factors among appendicitis patients in ethiopia, 2025

Author	Year	Selection (0–5)	Comparability (0–2)	Outcome (0–3)	Total NOS Score (0–10)
Chakebo D., et al.	2017	4	2	3	9
Admasu A., et al.	2020	4	2	3	9
Abebe D., et al.	2019	4	2	3	9
Jemal A., et al.	2018	3	2	2	7
Yonas Y., et al.	2019	4	2	3	9
Wolde M., et al.	2021	4	2	3	9
Zinabu A., et al.	2016	4	2	3	9
Hana G., et al.	2021	4	2	3	9
Tadiwos J., et al.	2014	3	1	2	6
Selamawit S., et al.	2021	4	2	2	8
Chewaka L., et al.	2024	4	1	2	7
Lambamo L., et al.	2018	4	2	2	8
Rocho A., et al.	2014	4	2	2	8
Mahlet A., et al.	2020	4	1	2	7
Mohammed Y., et al.	2022	4	1	2	7
Tekalign A., et al.	2019	4	2	2	8
Bechura B., et al.	2018	4	2	3	9
Bayisa H., et al.	2018	4	2	2	8

### Statistical analysis and synthesis

Data extracted from the eligible studies were compiled using Microsoft Excel and analyzed using STATA version 14. A random-effects model (DerSimonian and Laird method) was used to account for between-study variability. Heterogeneity across studies was assessed using the I² statistic and Cochran’s Q test. I² values of < 25%, 25–50%, and > 50% were interpreted as indicating low, moderate, and substantial heterogeneity, respectively [40]. Meta-regression was conducted using sample size and publication year as covariates to explore potential sources of heterogeneity. Publication bias was assessed using Egger’s test and visual inspection of funnel plots. When evidence of publication bias was detected, the trim-and-fill method was applied to adjust the pooled estimates. Subgroup analyses were conducted by region, time period and type of hospitals to explore variations in effect sizes. Subgroup analysis by study design was not conducted due to the inclusion of only one cohort study among the 18 eligible studies, limiting meaningful stratification. All results are presented with corresponding 95% confidence intervals (CIs), and p-values are interpreted with appropriate caution, emphasizing clinical relevance in addition to statistical significance. For the meta-analysis of predictors, adjusted odds ratios (AORs) were extracted from all included primary studies. Only adjusted estimates were included in the pooled analysis to enhance comparability and reduce potential bias from unadjusted associations.

## Results

### Study selection of included study

**Fig. 1:**
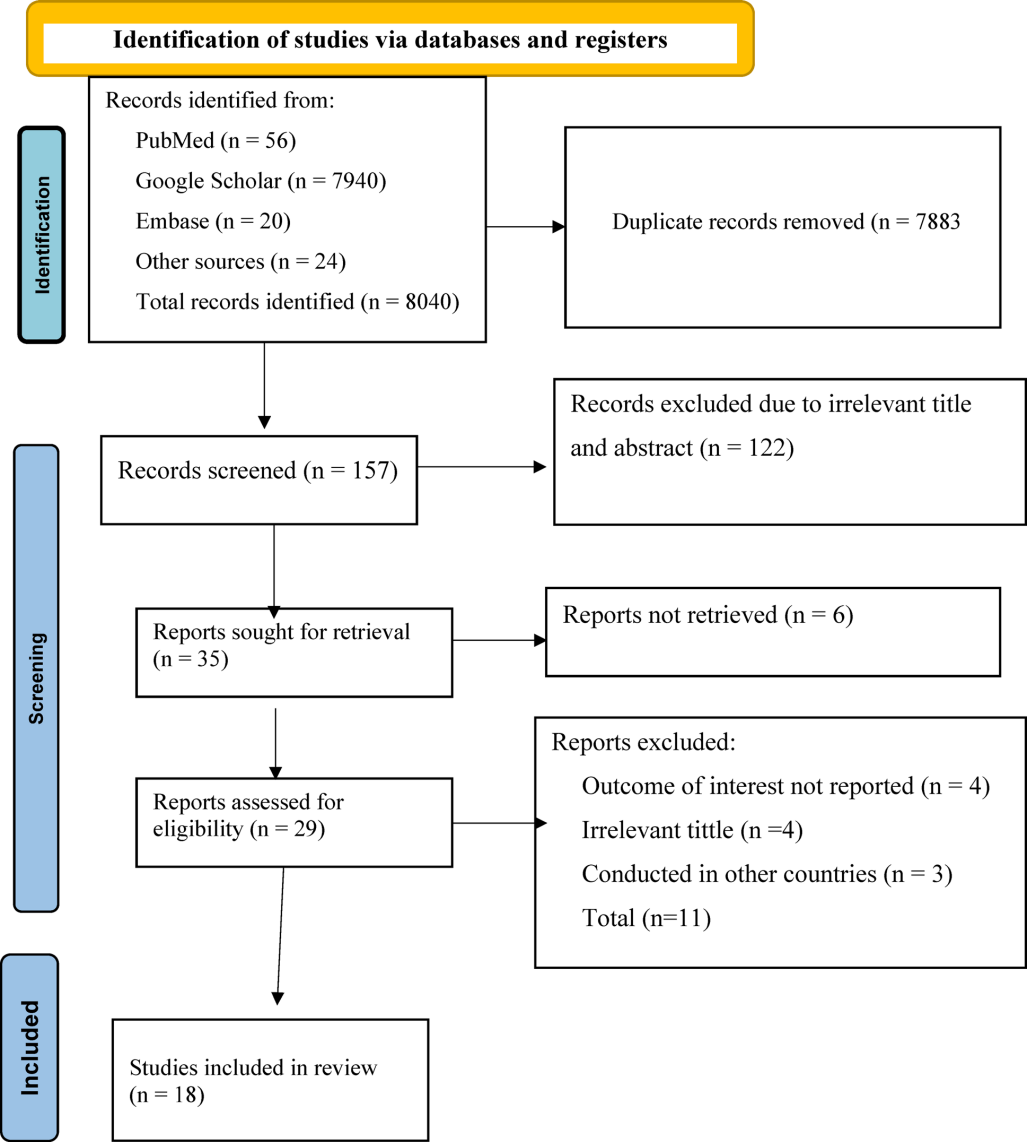
PRISMA 2020 flow diagram illustrating the study selection process for the systematic review and meta-analysis on unfavorable management outcomes of appendicitis in Ethiopia, 2025

From an initial pool of 8,040 records identified through a comprehensive search of major databases and grey literature, 7,883 duplicates records were removed. After screening title and abstract, 122 records were excluded based on irrelevance to the study objectives, additionally five full text articles could not be retrieved, and eleven studies were excluded due to absence of relevant outcome data. Ultimately, 18 studies involving a total of 7,442 participants were included in the final meta-analysis (Fig. 1).

### Characteristics of included studies

The majority of the included studies employed a cross-sectional design, with only one study using a cohort design. Geographically, the studies represented diverse regions of Ethiopia, including Oromia, Amhara, Sidama, Addis Ababa, and Central Ethiopia. The prevalence of unfavorable management outcomes reported in the included studies showed considerable variation, ranging from 1.4 to 31.9% [20, 25] (Table 2). This variation highlights potential differences in sample size, settings, or definitions of complications. Based on these studies, the pooled prevalence of unfavorable management outcomes among appendicitis patients in Ethiopia was subsequently estimated using meta-analytic techniques, with further analyses conducted to explore factors contributing to the observed variability.

Outcome Definition = Postoperative complications reported by the study (including but not limited to: fecal fistula, pneumonia, wound dehiscence, mortality, ileus, wound infection, sepsis, hernia, intra-abdominal fluid collection).

**Table 2:** Descriptive summary of the eighteen studies included in the systematic review and meta-analysis, reporting the prevalence and predictors of unfavorable management outcomes among appendicitis patients in ethiopia, 2025

Author	Year	Region	Study design	Sample size	Case	Proportion
Chakebo D., et al. [22]	2017	Oromia	Cross-sectional	382	39	10.2%
Admasu A, et al. [15]	2020	Amhara	Cross-sectional	310	31	10%
Abebe D., et al. [23]	2019	Amhara	Cross-sectional	169	45	26.6%
Jemal A…,et al. [20]	2018	Oromia	Cross-sectional	282	4	1.4%
Yonas Y., et al. [18]	2019	Oromia	Cross-sectional	245	35	14.31%
Wolde M., et al. [21]	2021	Amhara	Cross-sectional	300	36	12%
Zinabu A., et al. [24]	2016	Oromia	Cross-sectional	140	21	15%
Hana G., et al. [16]	2021	A.A	Cohort	227	9	3.8%
Tadiwos J., et al. [25]	2014	Oromia	Cross-sectional	182	58	31.9%
Selamawit S., et al. [17]	2021	Amhara	Cross-sectional	300	51	17%
Chewaka L., et al. [26]	2024	Sidama	Cross-sectional	336	17	5.1%
lambamo L.,et al. [41]	2018	Oromia	Cross-sectional	201	34	16.9%
Rocho A., et al. [42]	2014	Oromia	Cross-sectional	256	75	29.30%
Mahlet A., et al. [43]	2020	Amhara	Cross-sectional	119	6	5%
Mohammed Y., et al. [44]	2022	Amhara	Cross-sectional	148	6	4.10%
Tekalign A., et al. [45]	2019	CER	Cross-sectional	82	12	14.60%
Bechura B., et al. [46]	2018	Oromia	Cross-sectional	363	25	6.90%
Bayisa H., et al. [47]	2018	Oromia	Cross-sectional	130	17	13%

NB: CER = Central Ethiopia region, A.A = Addis Ababa

### Prevalence of unfavorable management outcomes in appendicitis patients

In this systematic review and meta-analysis, the pooled prevalence of unfavorable management outcomes among appendicitis patients in Ethiopia was estimated to be 12.71% (95% CI: 9.32% − 16.09%; *p* < 0.001), indicating a notable clinical concern.

**Fig. 2:**
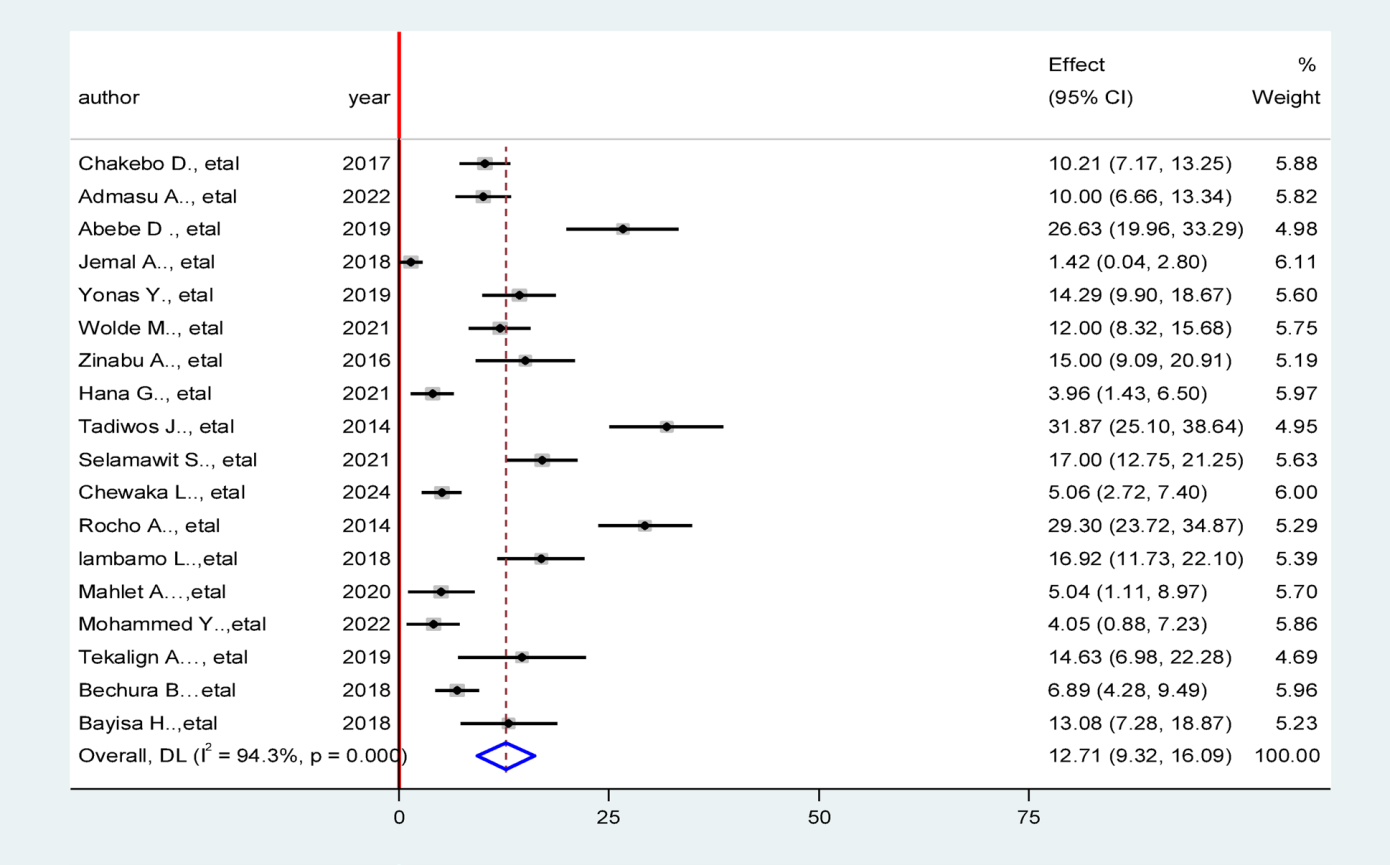
Forest plot showing the pooled prevalence of unfavorable management outcomes among appendicitis patients in Ethiopia, 2025

Marked heterogeneity was observed across studies (I² = 94.3%, *p* < 0.001), with Cochran’s Q test (Q = 297.08; df = 17; *p* < 0.001) and a between-study variance (Tau² = 48.2970) this substantial heterogeneity suggests potential differences in study populations, hospital settings, or outcome assessment methods. These findings warrant cautious interpretation of the pooled estimate and further exploration of sources of heterogeneity (Fig. 2).

### Types of unfavorable management outcomes

The analysis further stratified the types of unfavorable outcomes reported in the literature. Wound infection was the most common reported unfavorable management outcome, with pooled prevalence of 7.25% (95% CI: 4.94–9.58%), followed by paralytic ileus 2.5% (95% CI: 1.37–3.63%), pneumonia 1.64% (95% CI: 0.82–2.46%), fecal fistula 1.00% (95% CI: 0.48–1.52%), intra-abdominal fluid collection (0.8%, 95% CI: 0.28–1.32%), and in-hospital mortality 0.57% (95% CI: 0.19–0.94%) (Table 3).

**Table 3:** Types of unfavorable management outcomes among appendicitis patients in ethiopia, 2025

No	Types of unfavorable management outcome among appendicitis	Pooled prevalence with 95%CI
1	Wound infection	7.25(4.94–9.58)
2	Pneumonia	1.64(0.82–2.46)
3	Intra-abdominal fluid collection	0.8(0.28–1.32)
4	Fecal fistula	1.00(0.48–1.52)
5	Paralytic ileus	2.5(1.37–3.63)
6	Death	0.57(0.19–0.94)

### Region based Sub-group analysis

**Fig. 3:**
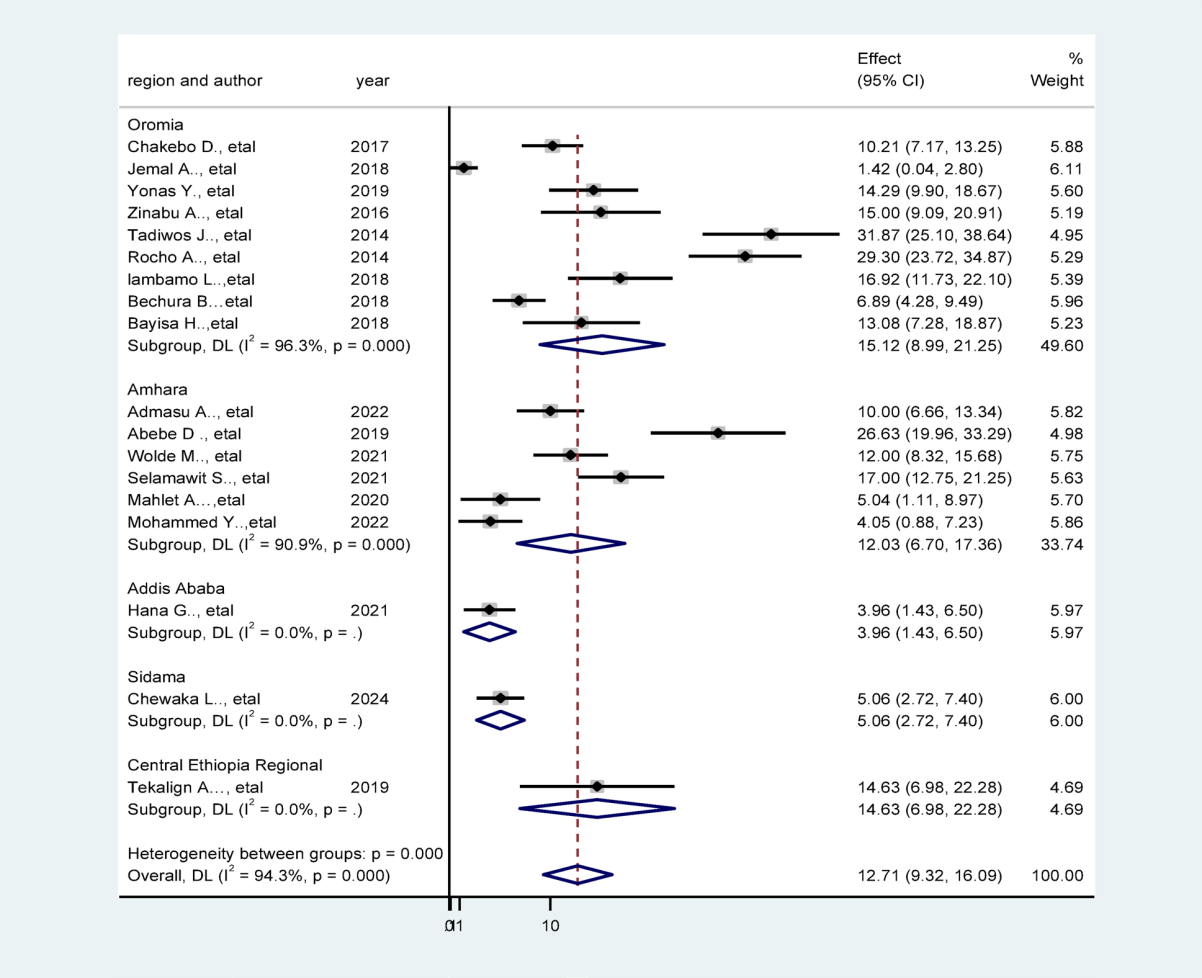
Forest plot of subgroup analysis showing prevalence of unfavorable management outcomes of appendicitis by regions, Ethiopia, 2025

Given the significant heterogeneity observed across the included studies, a region-based subgroup analysis was performed to explore potential sources of this variability. The Oromia Region exhibited the highest prevalence of unfavorable outcomes, with a rate of 15.12% (95% CI: 8.99–21.25%), accompanied by substantial within-group heterogeneity (I² = 96.3%, *p* < 0.001). This was followed by the Central Ethiopia Region, with a prevalence of 14.63% (95% CI: 6.98–22.28%). Other regions also demonstrated notable differences in prevalence estimates, with Amhara, Sidama, and Addis Ababa each displaying distinct patterns of heterogeneity. These findings suggest significant regional disparities in healthcare infrastructure, access to timely surgical care, efficiency of referral systems, and perioperative management practices. The observed heterogeneity reinforces the need for targeted, region-specific interventions to strengthen surgical service delivery and monitor outcomes more effectively (Fig. 3).

### Subgroup analysis by hospital type

**Fig. 4:**
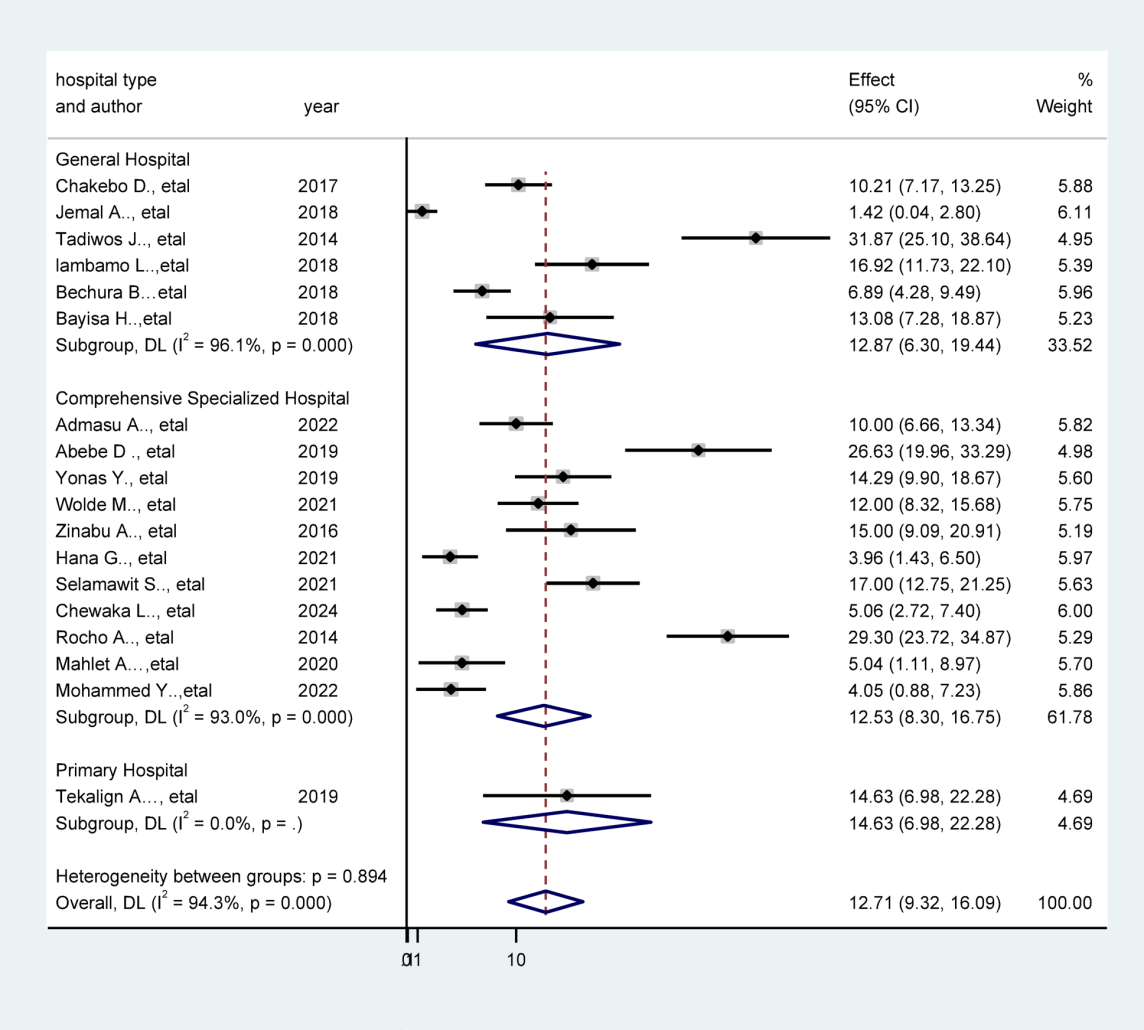
Forest plot of Subgroup analysis of showing the prevalence of unfavorable management outcomes of appendicitis by hospital types, Ethiopia, 2025

A subgroup analysis by hospital type revealed the highest prevalence of unfavorable management outcomes in primary hospitals (14.63%; 95% CI: 6.98–22.28%), followed by general hospitals (12.87%; 95% CI: 6.30–19.44%) and referral hospitals (11.10%; 95% CI: 7.21–15.00%). Comprehensive specialized hospitals (referral hospitals), which are equipped with more resources and specialized personnel, demonstrated lower rates of unfavorable outcomes. This indicates that hospital capacity significantly impacts patient outcomes and underscores the importance of improving the quality of care in primary and general hospitals (Fig. 4).

### Subgroup analysis by time period

**Fig. 5:**
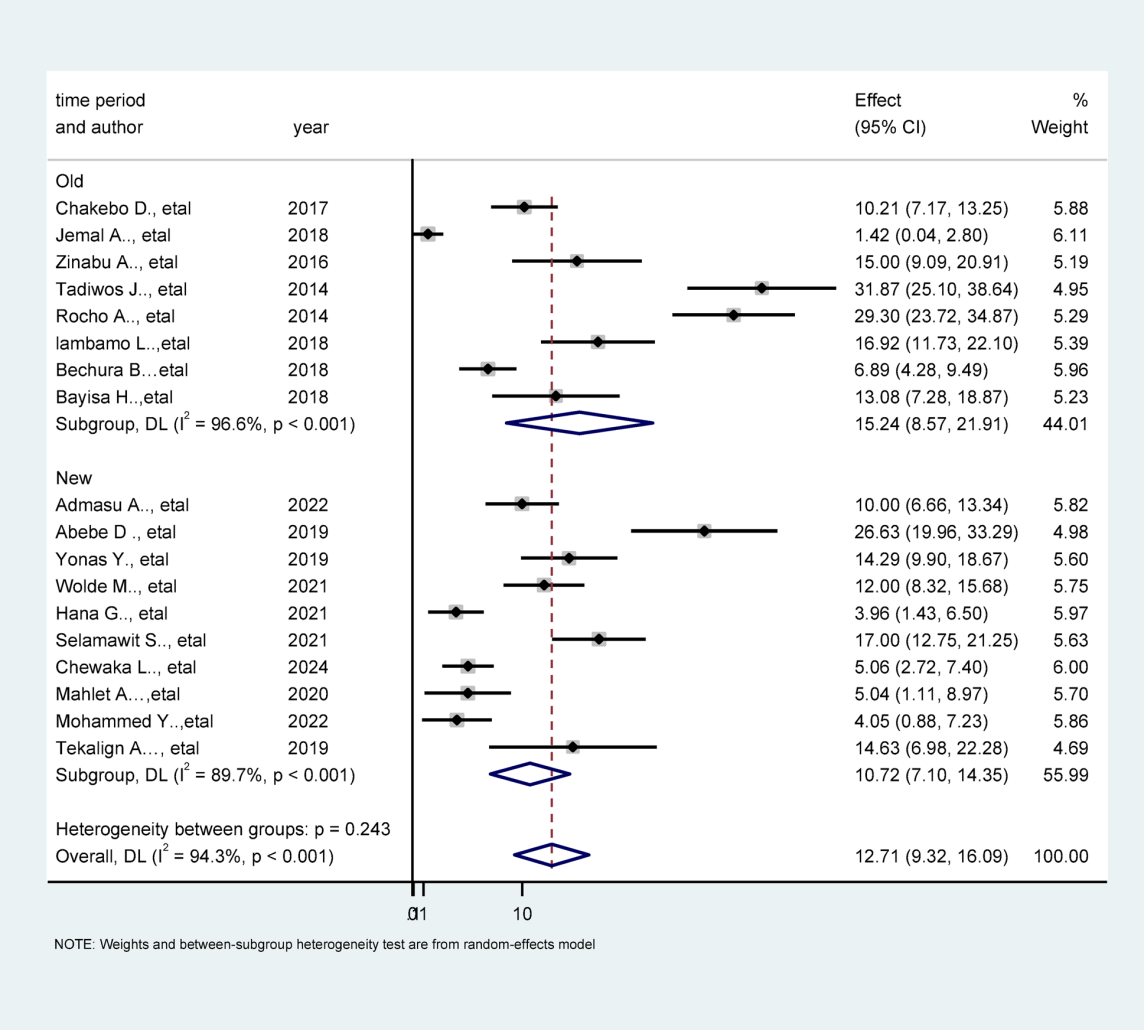
Forest plot of Subgroup analysis of showing the prevalence of unfavorable management outcomes of appendicitis by time period, Ethiopia, 2025

A subgroup analysis based on the publication period revealed a higher pooled prevalence of unfavorable management outcomes among studies published before 2019 (15.24%; 95% CI: 8.57–21.91%) compared to those published in 2019 and later (10.72%; 95% CI: 7.10–14.35%). This reduction over time may reflect improvements in clinical practices, diagnostic capacity, or resource allocation in more recent years (Fig. 5).

### Factors associated with unfavorable management outcome of appendicitis

In this systematic review and meta-analysis identified several factors significantly associated with unfavorable management outcomes in appendicitis patients in Ethiopia. These include prolonged duration of illness, right lower quadrant abdominal mass, presence of intraoperative abscess, length of postoperative hospital stay, elevated white blood cell count.

**Fig. 6:**
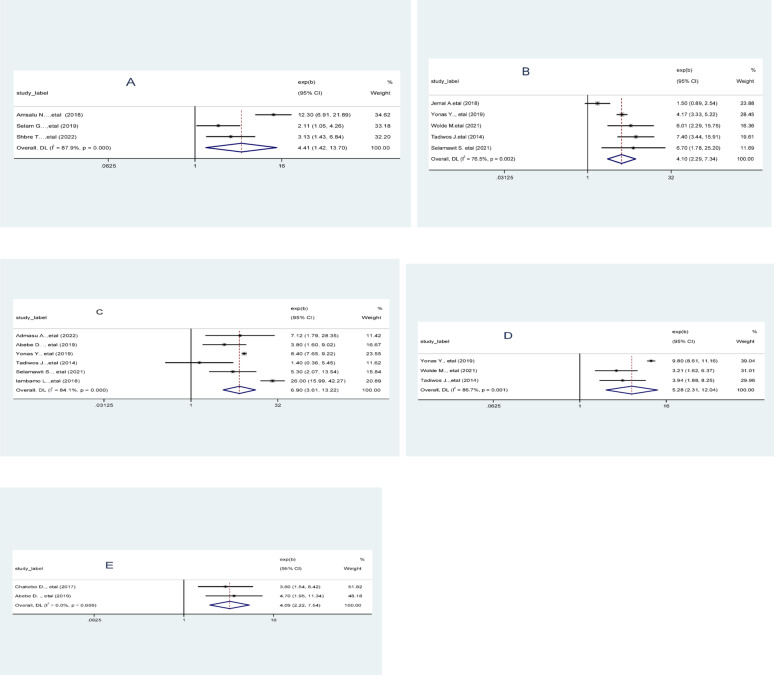
Pooled odds ratios (log scale) for predictors of unfavorable management outcomes of appendicitis in Ethiopia 2025: (**A**) Duration of illness, (**B**) Right lower abdominal quadrant mass, (**C**) intraoperative abscess, (**D**) Length of hospital stay, (**E**) Elevated WBC

Patients who had prolonged duration of illness were 4.41 times more likely to experience unfavorable outcomes (AOR = 4.41; 95% CI: 1.42–13.70). The presence of an RLQ mass increased the odds by four times (AOR = 4.10; 95% CI: 2.29–7.34). Patients with an intraoperative abscess had nearly seven times higher odds of poor outcomes (AOR = 6.90; 95% CI: 3.61–13.22). A longer hospital stay after surgery was also linked to worse outcomes (AOR = 5.28; 95% CI: 2.31–12.04). Additionally, elevated WBC count was associated with a fourfold increase in unfavorable outcomes (AOR = 4.09; 95% CI: 2.22–7.54). These findings were consistent across the included studies and underscore the importance of early diagnosis, prompt intervention, and effective infection control in improving appendicitis outcomes (Fig. 6).

### Meta-Regression

A meta-regression was conducted to explore whether publication year and sample size explained heterogeneity in the prevalence of unfavorable management outcomes. Neither publication year (*p* = 0.98) nor sample size (*p* = 0.15) showed a significant association. The overall model was not statistically significant (F [2, 15] = 1.20, *p* = 0.33), explaining only 2.22% of between-study variance. Considerable residual heterogeneity remained (I² = 69.14%), indicating that other unmeasured factors likely contribute to the observed variability.

### Publication bias

**Fig. 7:**
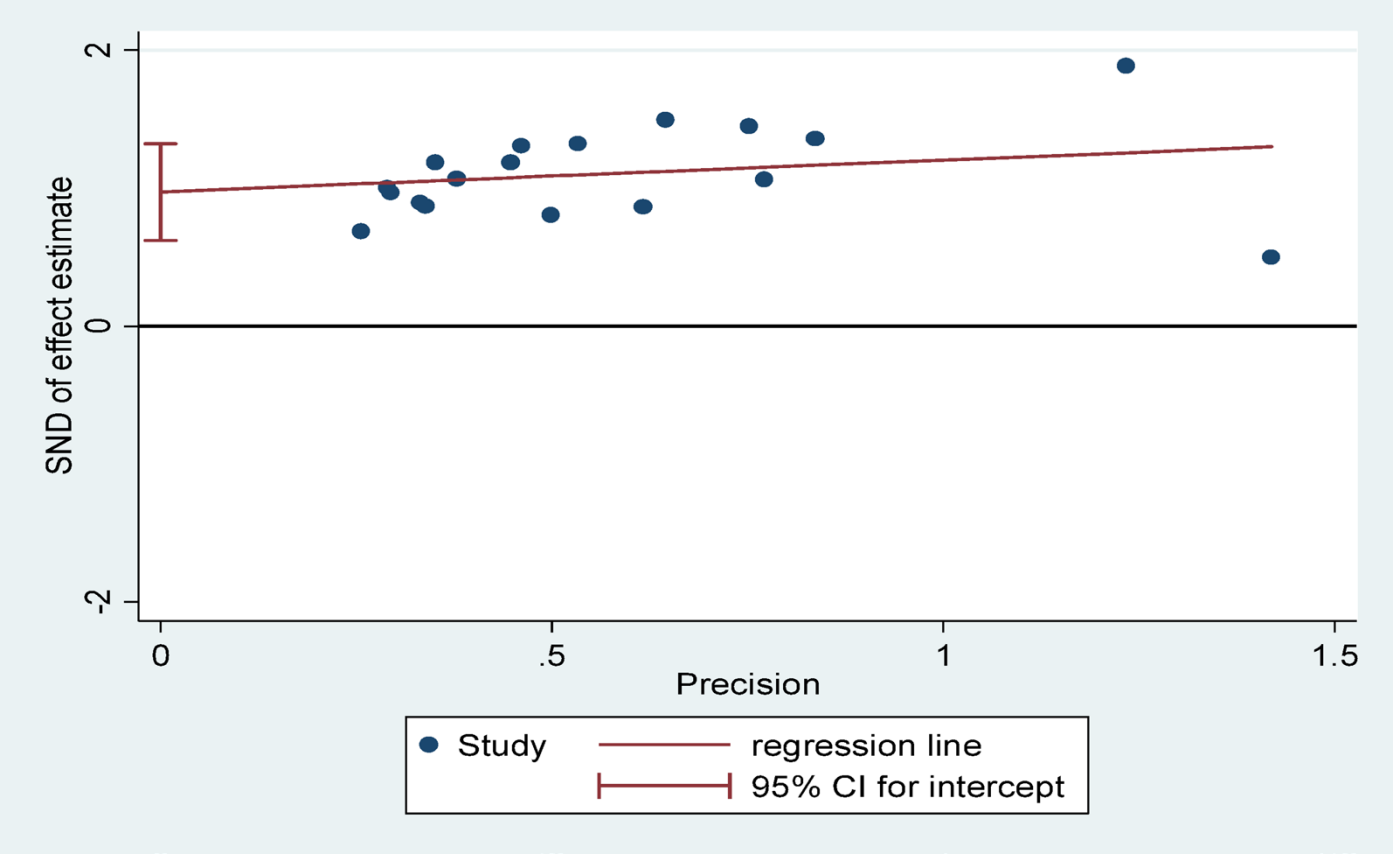
Egger regression test assessing publication bias for unfavorable management outcomes in appendicitis patients in Ethiopia, 2025

**Fig. 8:**
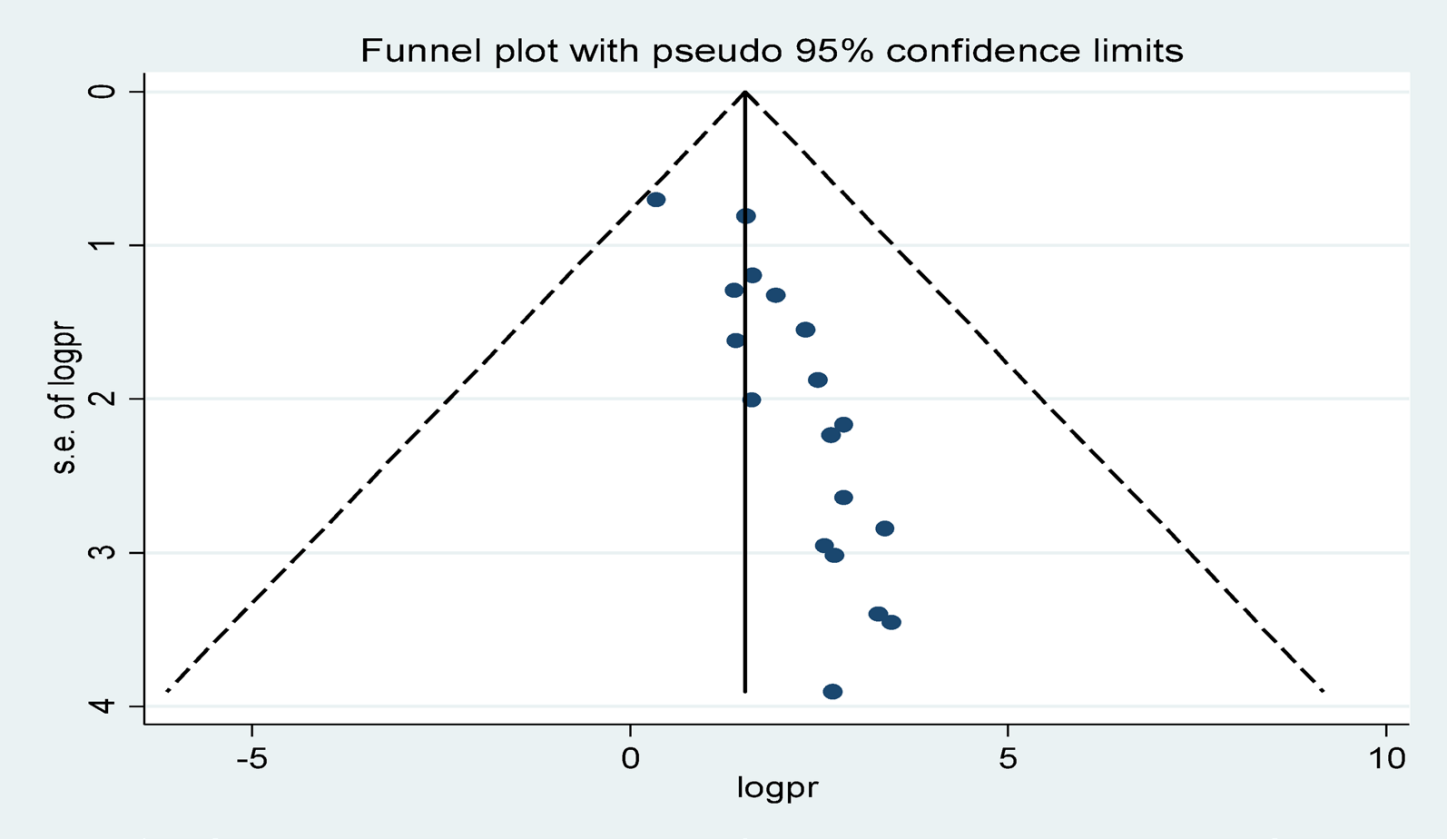
Funnel plot assessing publication bias for unfavorable management outcomes in appendicitis patients in Ethiopia, 2025

Publication bias was assessed through visual inspection of the funnel plot and Egger’s regression test. The funnel plot showed asymmetry, with a higher concentration of studies on the right side of the central effect line, suggesting that smaller studies with null or negative findings may be underrepresented. Egger’s test confirmed this, showing a significant small-study effect (bias = 0.9727, SE = 0.1647, *p* < 0.001). To adjust for this bias, the trim-and-fill method was applied, which estimated nine potentially missing studies. The pooled effect size decreased from 1.523 to 1.291 after adjustment, though it remained statistically significant. This suggests that while publication bias may have influenced the magnitude of the estimate, the overall direction and validity of the findings remain robust. Inclusion of both published and unpublished studies helped mitigate this bias, though residual bias cannot be entirely ruled out (Table 4, Figs. 7 and 8).

**Table 4:** Trim-and-fill regression test assessing publication bias for unfavorable management outcomes in appendicitis patients in Ethiopia, 2025

Iteration	Estimate	Tn	#to trim	Diff
1	1.523	150	7	171
2	1.351	163	9	26
3	1.291	164	9	2
4	1.291	164	9	0

### Sensitivity analysis

**Fig. 9:**
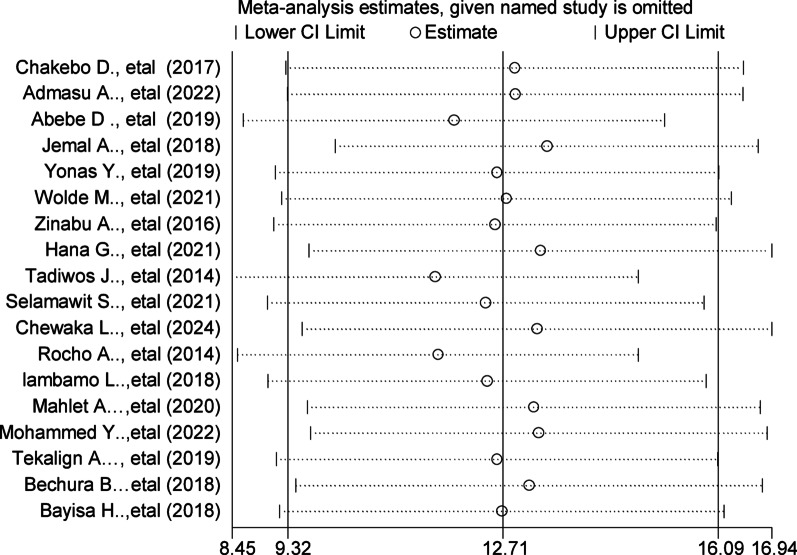
Sensitivity analysis assessing the influence of each individual study on the pooled prevalence in the systematic review and meta-analysis of unfavorable management outcomes among appendicitis patients in Ethiopia, 2025

A sensitivity analysis was conducted using the trim-and-fill method to evaluate the potential impact of publication bias. Although Egger’s test (*p* < 0.001) and visual inspection of the funnel plot indicated possible publication bias, the adjusted pooled prevalence estimate remained similar to the original. This suggests that the direction and magnitude of the effect were not substantially affected by the presence of missing studies, supporting the robustness of the main findings (Fig. 9).

## Discussion

### Prevalence of unfavorable management outcome among appendicitis patients

This systematic review and meta-analysis estimated a pooled prevalence of unfavorable management outcomes among appendicitis patients in Ethiopia at 12.71% (95% CI: 9.32% − 16.09%), indicating a moderate rate of postoperative complications. This finding provides important insight into the quality of appendicitis care and surgical outcomes in Ethiopia.

The prevalence identified in this study is comparable to those reported 9.7% in Sri Lanka [48], 10.6% in USA [49], 13.5% in Nigeria [50], 13.2% in Italy [51], suggesting potential similarities in population characteristics or healthcare service utilization. Conversely, it is notably higher than rates reported 0.5% in Israel [52], 3% in Canada [53], 4.8% in Finland [54], 6.4% in Bangladesh [55]. These lower figures may reflect better healthcare infrastructure, early diagnosis, and established perioperative protocols common in high-income countries. In contrast, the prevalence is lower than reports from Nigeria 15.9% [56], Uganda 20.6% [57], Finland 24.4% [58], South Africa 42.8% [59], where differences in health system capacity, case mix, or reporting standards may contribute to higher estimates.

### Subgroup analysis

Subgroup analysis revealed meaningful variation in unfavorable management outcomes across regions, hospital types, and study time periods. The highest prevalence was observed in the Oromia region, likely reflecting health system disparities, including limited surgical capacity, delayed referrals, and gaps in rural–urban healthcare access. These regional disparities are well-documented in National Health Service assessments and reflect uneven distribution of trained surgical personnel and diagnostic services [60]. Similarly, a higher prevalence of unfavorable outcomes was found in primary hospitals compared to general and referral hospitals. These differences likely reflect disparities in human resources, surgical infrastructure, and clinical protocols. Primary and general hospitals in Ethiopia often face shortages of surgical personnel, limited access to anesthesia services, and constrained diagnostic and postoperative care capacities. These systemic limitations may delay timely intervention or compromise postoperative monitoring, thereby increasing complication rates [61].

The time period subgroup analysis showed a lower prevalence of unfavorable outcomes in studies published from 2019 onward, suggesting improvements in surgical systems and care quality over time. National initiatives such as the Saving Lives through Safe Surgery (SaLTS) program (2016–2020) have contributed to expanding surgical infrastructure, standardizing protocols, and training mid-level surgical providers [62]. However, recent national evaluations still highlight challenges in consistent implementation. A 2025 nationwide assessment found that only 77% of public hospitals adhered to essential patient monitoring and diagnostic protocols despite adequate infrastructure, pointing to gaps in system readiness and quality enforcement [60].

Diagnostic delays remain a critical contributor to poor outcomes across all hospital levels. Many facilities lack timely access to imaging modalities such as ultrasound and CT scans, as well as basic laboratory services like white blood cell (WBC) counts. These limitations often force clinicians to rely solely on clinical signs, increasing the risk of misdiagnosis, delayed surgical intervention, and complications such as perforation or abscess formation. Rural and underserved areas face additional barriers, including weak referral systems and geographic inaccessibility, which further exacerbate delays. These challenges are consistent with international and national reports that identify diagnostic and surgical care gaps in low- and middle-income countries, including Ethiopia [63–65]. Strengthening diagnostic capacity, particularly in primary and general hospitals remains essential to improving early detection and reducing appendicitis-related morbidity.

### Types of unfavorable management outcome among appendicitis patient

Wound infection was the most frequently reported unfavorable management outcome in this review, with a pooled prevalence of 7.25%. This rate aligns with the surgical site infection (SSI) incidence commonly observed in low- and middle-income countries (LMICs), which typically ranges from 5 to 15% [37, 57, 66, 67]. SSIs remain a major postoperative complication worldwide, influenced by factors such as surgical technique, perioperative antibiotic use, and healthcare infrastructure quality [68]. Paralytic ileus 2.5% and pneumonia 1.64% were also notable complications, consistent with reports from similar resource-limited settings [37, 66]. Less frequent but clinically important unfavorable management outcome included fecal fistula 1.00%, intra-abdominal fluid collection 0.8% and death 0.57% [69–71]. These findings highlight the importance of improving infection control measures, perioperative care, and access to timely surgical intervention to further reduce the burden of unfavorable management outcomes in Ethiopia.

### Factors associated with unfavorable management outcome of appendicitis patients

This systematic review and meta-analysis identified several clinical predictors that were significantly associated with unfavorable management outcomes following appendicitis surgery in Ethiopia. Specifically, intraoperative appendiceal abscess, prolonged duration of illness before hospital arrival, longer hospital stays, right lower quadrant (RLQ) abdominal mass, and elevated white blood cell (WBC) count were statistically significant factors. These findings are consistent with previous studies both within Ethiopia and across other low- and middle-income countries (LMICs), highlighting systemic and clinical challenges commonly faced in resource-constrained settings.

Intraoperative appendiceal abscess was found to significantly increase the risk of poor postoperative outcomes, consistent with findings from studies in Uganda [57], and Ethiopia reports [15–18, 23, 25]. In LMICs, delays in diagnosis and treatment often result in perforated appendicitis or abscess formation, which leads to intra-abdominal contamination and increased surgical complexity. This contamination raises the risk of surgical site infections (SSI), which remains one of the most prevalent postoperative complications in these settings due to limitations in sterile techniques, antibiotic access, and perioperative care [72].

Delayed presentation, defined as prolonged symptom duration before hospital arrival, was also a significant predictor of unfavorable outcomes. Similar results have been reported in both Ethiopian studies [36, 37]. In many low-income settings, delayed healthcare-seeking is influenced by poor access to medical facilities, limited health literacy, economic constraints, reliance on traditional healers, and systemic delays in referral. These factors allow the disease to progress to more complicated stages, increasing the likelihood of perforation, abscess, or peritonitis, and ultimately leading to more invasive surgeries and higher complication rates [69].

Length of hospital stay was also significantly associated with unfavorable management outcomes. This is supported by studies from Ethiopia [18, 21, 25], in USA [73], and Singapore [74]. In LMICs, prolonged hospitalization may increase the risk of nosocomial infections due to overcrowded wards, inadequate infection control practices, and limited access to antibiotics or sterile surgical supplies [37].

Presence of right lower quadrant abdominal mass was significantly associated with unfavorable outcome of appendicitis this is in line with previous studies [17, 18, 21, 25]. This might be due to the scientific fact that the more tenderness of body parts is the most difficult surgical procedure. In many LMICs, the availability of imaging and diagnostic tools to assess such complications preoperatively is limited, further compounding the risk [63].

Patients with elevated WBC count (> 11,000 cells/nl) were more likely to experience postoperative complications. This association was also found in studies from Nepal [75] and Ethiopia [22, 23]. The possible explanation might be due to the fact that the presence of infections can trigger the body’s immune system and cause inflammation and tissue damage or delay wound healing.

### Clinical implications and health system context

Clinical Implications and Health System Context This review highlights systemic factors that contribute to poor appendicitis outcomes in Ethiopia. Inadequate access to imaging (e.g., ultrasound, CT), laboratory testing (e.g., WBC count), and trained surgical personnel in many public hospitals results in delayed diagnosis and complications such as abscess or sepsis [60]. Referral delays, long travel distances, and weak pre-hospital triage systems also worsen outcomes [65]. To address these issues, Ethiopia’s surgical health system should prioritize timely referral pathways, expand diagnostic infrastructure at primary and district hospitals, and implement infection control protocols. Furthermore, establishing nationwide surgical audits and perioperative quality improvement programs is essential to standardize care and reduce outcome disparities across regions [60, 61].

## Conclusion

This systematic review and meta-analysis demonstrated a moderately high pooled prevalence of unfavorable management outcomes among appendicitis patients in Ethiopia. Key factors associated with poor outcomes included intraoperative appendiceal abscess, delayed hospital presentation, prolonged hospital stay, presence of right lower quadrant abdominal mass, and elevated white blood cell count.

To address these challenges, a combination of public health and clinical interventions is recommended. Improving early diagnosis through community health education and referral system strengthening could reduce delays in care-seeking. Incorporating clinical indicators such as palpable abdominal mass and leukocytosis into preoperative risk assessments may support better clinical decision-making. Additionally, reinforcing infection prevention measures, expanding surgical infrastructure—especially at primary and district hospitals—and standardizing perioperative protocols are essential to improving outcomes in resource-constrained settings. These findings offer actionable insights for clinicians, hospital managers, and policymakers working to enhance surgical care quality in Ethiopia.

Future Directions: High-quality, multicenter prospective cohort studies are warranted to validate these results, improve generalizability, and inform tailored evidence-based interventions applicable to Ethiopia and similar low- and middle-income countries.

### Limitations

This systematic review and meta-analysis has several limitations. First, although a standardized operational definition of “unfavorable management outcomes” was applied, variations in outcome definitions and reporting across the included studies may have introduced clinical heterogeneity. Second, most studies were cross-sectional, which limits the ability to establish causality or temporal relationships between predictors and outcomes. Moreover, only one of the 18 included studies employed a cohort design, making it infeasible to conduct subgroup analysis by study design as originally intended.

Third, while all studies met the minimum quality threshold using the Newcastle–Ottawa Scale, this tool may not fully capture surgical-specific quality domains, and variability in study quality could have influenced our pooled estimates. Fourth, although subgroup and meta-regression analyses were performed, limited reporting in the primary studies prevented exploration of other potentially important factors such as surgeon experience, disease severity, or perioperative protocols.

Fifth, significant publication bias was detected through Egger’s test and funnel plot asymmetry. Although the trim-and-fill method confirmed the robustness of the results, residual bias cannot be entirely excluded. Sixth, our review was limited to English-language publications, and while the inclusion of grey literature enhanced national coverage, it may have introduced variability due to limited peer review. Lastly, as a secondary analysis, the findings are inherently dependent on the quality and completeness of the original studies.

Despite these limitations, the review provides important insights into the burden and determinants of unfavorable appendicitis outcomes in Ethiopia and can inform future clinical practice, research, and health system planning.

## Data Availability

The datasets used and/or analyzed during the current study are available from the corresponding author upon reasonable request.

## Abbreviations

AA: Addis Ababa
AOR: Adjusted Odd Ratio
LMIC: Low and Middle Income Countries
SSI: Surgical Site Infection
CRE: Central Region of Ethiopia
CI: Confidence Interval
WBC: White blood cell
